# *Pseudomonas aeruginosa* and related antibiotic resistance genes as indicators for wastewater treatment

**DOI:** 10.1016/j.heliyon.2024.e29798

**Published:** 2024-04-20

**Authors:** Alariqi Reem, Siham Almansoob, Ahmed M. Senan, Aditya Kumar Raj, Rajesh Shah, Mukesh Kumar Shrewastwa, Jay Prakash Prasad Kumal

**Affiliations:** a Medical Laboratory Department, Faculty of Medical Sciences, Amran University, Yemen; b International department, Changsha medical university, Changsha, Hunan, 410000, China; c Department of Basic Pharmaceutical Sciences, Faculty of Pharmacy, Suleyman Demirel University, Isparta, 32260, Turkey; d Department of Physiology, National Medical College, Birgunj, Nepal; e Department of Microbiology, Nepalgunj Medical College, Chisapani, Banke, Nepal; f Department of Biochemistry, Nepalgunj Medical College, Kohalpur, Banke, Nepal; g Department of Biochemistry (IMS & SUM hospital), SOA, deemed to be University, Bhubaneswar, India; h Department of Human Anatomy, Nepalgunj Medical College, Nepalgunj, Nepal

**Keywords:** *Pseudomonas aeruginosa*, Antibiotic resistance genes, UV chlorine, Catalytic oxidation, Fenton reaction, Ozonation

## Abstract

This review aims to examine the existence of *Pseudomonas aeruginosa (P*. *aeruginosa) a*nd their antibiotic resistance genes (ARGs) in aquatic settings and the alternative treatment ways. *P. aeruginosa* in a various aquatic environment have been identified as contaminants with impacts on human health and the environment. *P. aeruginosa* resistance to multiple antibiotics, such as sulfamethoxazole, ciprofloxacin, quinolone, trimethoprim, tetracycline, vancomycin, as well as specific antibiotic resistance genes including *sul1, qnrs, blaVIM, blaTEM, blaCTX, blaAIM-1, tetA, ampC, blaVIM.* The development of resistance can occur naturally, through mutations, or via horizontal gene transfer facilitated by sterilizing agents. In addition, an overview of the current knowledge on inactivation of *Pseudomonas aeruginosa* and ARG and the mechanisms of action of various disinfection processes in water and wastewater (UV chlorine processes, catalytic oxidation, Fenton reaction, and ozonation) is given. An overview of the effects of nanotechnology and the resulting wetlands is also given.

## List of abbreviations

UV: Ultra violet
ARGs: Antibiotic resistance genes
CF: Cystic fibrosis
MDR: Multidrug-resistant
EDNA: Extracellular DNA
IDNA: Intracellular DNA
WWTPs: Wastewater treatment plants
HGT: Horizontal gene transfer
ABR: Antibiotic resistance bacteria
MDRPA: Multi-drug-resistant *Pseudomonas aeruginosa*
AMSB: Antimicrobial susceptible bacteria
GIs: Genomic islands

## 1 Introduction

Our planet, referred to as the "blue planet,” is predominantly covered by water, however, only a small fraction (2.5 %) of this vast resource is freshwater. In light of growing challenges such as changing lifestyles, global water pollution, and declining water quality [1], the critical importance of clean and safe water cannot be overstated. Unfortunately, rapid industrialization, urban expansion, and intensified agricultural practices have led to a surge in toxic wastewater production, posing severe threats to both human health and the environment [2]. Water can harbor a variety of harmful inorganic, organic, and biological pollutants, some of which are carcinogenic [3]. Despite significant advancements in the 20th century, including filtration and chlorination, it is disheartening that more than a billion people worldwide still lack access to clean drinking water [4].

In the 21st century, the challenges in managing water quality have become even more pressing, particularly in densely populated urban areas and regions like the Middle East, where effective water management remains a struggle [5]. The increasing threat of pollutants to water infrastructure is a matter of great concern, as treatment systems often struggle to efficiently remove the contaminants they produce, posing significant health risks to both humans and the environment [6].

Waterborne diseases remain a leading global cause of mortality, with unsafe and contaminated water contributing to hundreds of thousands of deaths, primarily among children [7].

*Pseudomonas aeruginosa (P*. *aeruginosa)* is a bacterium that can contaminate various types of water, including drinking water [8]. According to one study, eight isolates of *Pseudomonas* spp. of the *Pseudomonas putida and fluorescent* species were found in the consumption water. Therefore, in times of risk, *Pseudomonas* could participate in the emergence of antibiotic resistance by drinking water [9]. Its ability to form biofilms in plumbing (such as showerheads, faucets, etc.) is more important than its presence in the supply system or drinking water treatment because *P*. *aeruginosa* can live in distilled or deionized water [10]. In addition, tap water from hospitals [11]. Bronchoalveolar lavage fluid and water for rinsing in microbiological surveillance [12] Hospital water system [13]. In quantities ranging from 10/100 mL to over 1000/100 mL in natural waters such as lakes and rivers [14]. Water from a rural well [15]. According to the taxonomic designation, *Pseudomonas* and *Legionella* were the most detected categories of opportunistic pathogenic bacteria in both treated and untreated rainwater [16]. *P. aeruginosa*, a Gram-negative bacterium, is widespread across diverse ecological niches in water, soil, and various organisms, including humans [17]. Its multidrug-resistant (MDR) strains are a significant concern in nosocomial infections, including urinary tract infection and severe respiratory particularly affecting individuals with cystic fibrosis (CF), infection in burns, open wounds [18,19]. Foot infection in diabetics and others with poor microvascular circulation; ear infection, particularly otitis externa and chronic suppurative otitis media, which is related with tissue damage and water obstruction; and keratitis, which is associated with prolonged contact lens usage and contaminated contact lenses. Other rare but deadly infections include endocarditis occurring in people with or without injectable drug use, and meningitis linked with penetrating trauma to the head, insertion of a CNS shunt (such as a ventriculoperitoneal (VP) shunt), or post-neurosurgical operations [20,21].

Multidrug resistance has been increased all over the world that is considered a public health threat. Several recent investigations reported the emergence of multidrug-resistant bacterial pathogens from different origins that increase the necessity of the proper use of antibiotics [22]. Besides, the routine application of the antimicrobial susceptibility testing to detect the antibiotic of choice as well as the screening of the emerging MDR strains [23]. The escalating resistance of *P. aeruginosa* to conventional antibiotics necessitates innovative eradication methods [24].

## 2 Virulence determinants of *P. aeruginosa*

### 2.1 Secretion systems

The Type 1 secretion system (T1SS) transfers the alkaline protease AprA, while the Type 2 secretion system (T2SS) controls the secretion of multiple virulence factors, including exotoxin A (ToxA), proteases LasA and LasB, and the hemolytic phospholipase C (PlcH). The Type 3 secretion system (T3SS) functions as a needle-like nanomachine that delivers toxic effectors – ExoS, ExoT, ExoU, and ExoY – into the target host cell [25]. *P. aeruginosa* exhibits remarkable survival mechanisms against antimicrobial agents due to essential protein secretion systems, such as the type VI secretion system (T6SS), which playing a role in virulence and antibacterial competition [26]. Furthermore, the functional annotation of hypothetical proteins (HPs) may unveil new targets to enhance *P. aeruginosa* treatment and investigation [27,28].

## 3 Significance biofilm formation in *P. aeruginosa*

The establishment of a microbial biofilm relies on the creation of a matrix consisting of extracellular polymeric molecules that embed the bacteria together into a strong colony. The matrix is composed of three exopolysaccharides (Pel, Psl, and alginate), as well as extracellular DNA and proteins. These bacterial biofilm populations are usually resistant to antibiotic treatment and human immunity [21].

Their contamination potential extends to various water types, including drinking water, with specific *Pseudomonas* spp. Isolates, such as *P. Putida* and *P. fluorescens*, posing a potential risk for antibiotic resistance emergence [8,9]. Biofilm formation, a crucial defense mechanism against antibacterial drugs, is prevalent in aquatic environments, contributing to persistent infections and increased morbidity [29]. *P. aeruginosa's* adaptability to diverse water sources, its presence in plumbing systems, and its identification in various natural water bodies emphasize the need for effective water management and treatment [[10], [11], [12], [13], [14], [15], [16],^30^].

## 4 Antibiogram of *P. aeruginosa*

*P. aeruginosa* shows resistance to a range of antibiotics, including aminoglycosides, β-lactams and quinolones. Antibiotic resistance in *P. aeruginosa* is mostly due to acquired and intrinsic mechanisms, including poor membrane permeability, antibiotic resistance genes, and active efflux pumps. *P. aeruginosa's* outer membrane proteins (oprL) play an important role in antibiotic and antiseptic resistance. ESBL genes encode extended β-lactamases (ESBLs), which cause resistance to β-lactam antibiotics like penicillin and cephalosporins. The most prevalent ESBL genes associated with *P. aeruginosa* are ^bla^CTX-M and ^bla^TEM [31].

**Fig. 1:**
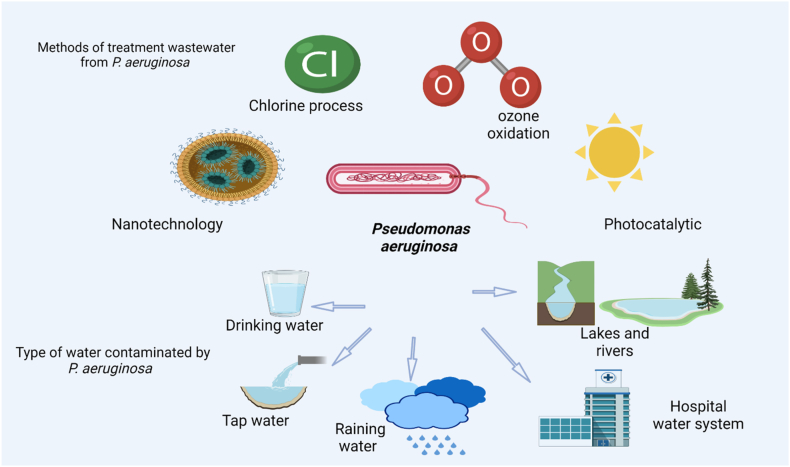
Types of water contamination of *P. aeruginosa*.

The widespread contamination of water bodies and the emergence of antibiotic-resistant bacteria have become significant global public health concerns, highlighting the urgency to develop advanced technologies for enhancing wastewater treatment standards [32] (Fig. 1). Antibiotic resistance has escalated to a critical global health issue in the twenty-first century, leading to a decline in the efficacy of antibiotics for treating infections, as recognized by the World Health Organization [33].

Antibiotic resistance genes (ARGs) are detected in both extracellular and intracellular DNA (eDNA and iDNA), with eDNA originating from bacterial cell lysis and active secretion from live bacteria [34]. These genes have been found in various aquatic environments, often involving mobile genetic components such as plasmids, integrons, and transposons [35,36]. Human and animal waste is the primary source of antibiotic dissemination into the environment, containing significant amounts of unmetabolized antimicrobials [37]. The widespread use of antibiotics in households results in their presence in wastewater, making municipal wastewater treatment plants (WWTPs) major contributors to ARGs and antibiotic-resistant bacteria (ARB) in the environment [38].

WWTPs have been identified as hotspots for horizontal gene transfer (HGT), enabling the widespread transmission of ARGs and serving as potential storage and ecological reservoirs for antibiotic resistance [39]. Antibiotics’ environmental persistence has been discovered in water resources, with adverse effects on environmental health, impacting microbial structure and development in ecology [[40], [41], [42]]. ARGs are now recognized as environmental pollutants that can spread through human and animal sources, affecting drinking water supplies and natural ecosystems [39,43,44]. Their persistence in streams and rivers is observed even after being released from hospital wastewater treatment plants (HWWTP) [45,46].

To safeguard the aquatic environment and human health, it is imperative to develop innovative methods for assessing the environmental risks associated with the spread both antimicrobial susceptible bacteria (AMSB) and antimicrobial-resistant bacteria (AMRB) in the aquatic environments [47].

This comprehensive review seeks to address a critical concern in water quality management-the presence and persistence of *P. aeruginosa*, a bacterium recognized by the World Health Organization for its potential threat to human health. We delve into the persistence of *P. aeruginosa* on hospital surfaces and its alarming resistance to antimicrobial agents. By examining methods to decontaminate water sources from this pathogen, our objective is to provide insights into effective strategies that can safeguard both public health and the environment.

## 5 *Pseudomonas aeruginosa* antibiotic resistance genes (ARGs)

*P. aeruginosa*, a bacterium with increasing antibiotics resistance, is a key concern in hospital wastewaters, identified as a significant source of antibiotics, antibiotic resistant bacteria (ARBs), and ARGs in the environment [48]. Resilient on hospital surfaces, *P*. *aeruginosa* contributes to the global presence various ARGs such as *sul1, qnrs, blaVIM, blaTEM, blaCTX, blaAIM-1, tetA, ampC, blaVIM* [49,50]. The potential for aquatic ecosystems to facilitate the interaction between indigenous bacteria and antibiotic-resistant ones, introduced via wastewater discharges, may lead to the transmission of ARGs to humans and animals through drinking water [51].

**Fig. 2:**
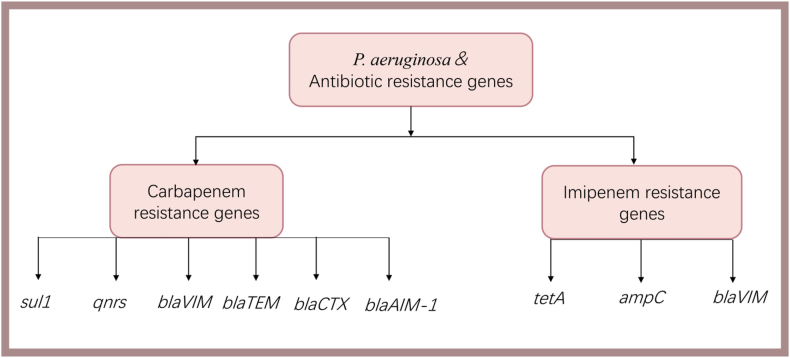
Main carbapenems and imipenem genes known to be involved in increase *P. aeruginosa* antibiotic resistance.

Even with disinfection measures in place to inactivate ARBs, a notable proportion of ARGs may persist, raising concerns about their transmission [52]. Vertical transmission plays a crucial role in the dissemination of acquired antibiotic resistance by *P*. *aeruginosa* through water [53]. Carbapenems, a vital class of beta-lactams for severe infections, encounter particular challenges with resistance in *P. aeruginosa,* further complicated by limited global approval for veterinary medicine use [54,55]. The detection of the *bla*_*AIM-1*_ gene for carbapenems resistance in wastewater samples from diverse sources, including healthcare and non-healthcare streams, as well as river water underscores the potential environmental impact [56] (Fig. 2).

The transmission and mobility of acquired genes, especially those encoding carbapenemases, involve various mobile genetic elements like plasmids, integron gene cassettes, transposons, and genomic islands. These components can migrate between genomes, exhibiting both intracellular and intercellular mobility through mechanisms such as transformation, conjugation, and transduction [57].

## 6 The link between the accessory genome and resistance dissemination in *P. aeruginosa*

Certain stains of *P. aeruginosa*, exemplified by Gene, exhibit remarkable adaptability to specific environmental conditions, resulting in the formation of an accessory genome. This genome encompasses a diverse array of genetic elements, including genomic islands (GIs), transposons, integrons, insertion sequences, prophages, plasmids, and integrative and conjugative elements (ICEs). Additionally, the overall genome of PA34 consists of 6.8 Mbp chromosome and two plasmids of 95.4 Kbp (pMKPA34-1) and 26.8 Kbp (pMKPA34-2), collectively encoding 1213 genes that contribute to a sizable auxiliary genome. Notably, these unique genes are associated with various attributes, including phage integrase, transposons, and both metal and antibiotic resistance.

Among the genomic islands (GIs), approximately 24 have been predicted within the entire chromosome, with two integrated into distinct locations. Remarkably, eleven of these GIs either replace pathogenic genes or bear virulence factors. The inclusion of the aminoglycoside resistance gene (AAC (3)-IId) within a bacteriophage highlights the intricate nature of resistance mechanisms in *P. aeruginosa* [58]. The larger genome size of *P. aeruginosa* may facilitate horizontal gene transfer (HGT) across diverse organisms due to reduced effects of codon bias. The elevated guanine cytosine (GC) content of *P. aeruginosa* also contributes to the assimilation of foreign DNA. However, genes and elements acquired through HGT may display a lower GC content due to genetic drift [59].

Emerging evidence suggests that mutational events play a significant role in the development of antibiotic resistance (AR) in *P. aeruginosa*. The proliferation of mobile genetic elements (MGEs) safeguarding AR genes adds to the complexity of the accessory genome, potentially carrying significant implications for public health [60].

## 7 Methods of treatment

### 7.1 Nanotech advances in water treatment: antibacterial impact

Over the last few decades, research in nanomaterial and nanotechnology has yielded a new understanding of fundamental processes and introduced revolutionary breakthroughs across a variety of traditional fields, including wastewater treatment. Nanotechnology is being explored as a potentially useful technology, and it has already shown excellent progress in multiple areas [61]. It is anticipated that the highly modular, multifunctional, and efficient processes of nanotechnology will provide powerful, high-performance, and affordable solutions for water and wastewater treatment [62]. Nanomaterial is generally defined as materials that are smaller than 100 nm in at least one dimension and possess high reactivity, functionalization, a large specific surface area, and size-dependent properties that make them suitable for water purification [63]. Certain nanomaterial can be used as disinfectants due to their antibacterial properties, reducing the risk of toxic disinfection byproducts (DBPs) forming during the typical disinfection process [64]. Various types of metal nanoparticles (NPs) have been found to cause multiple injuries to bacterial cells, such as disintegration of peptidoglycan, rupture of the cell membrane leading to the leakage of intracellular contents, release of reactive oxygen species (ROS) resulting in bacterial cell death, and the harmful effects of heavy metal ion release on cellular components [65]. Research trends indicate a growing interest in exploring nanoparticles to treat antibiotic-resistant bacteria [66]. Additionally, for economic and unspecified health reasons, the nanotechnology is expected to become increasingly important in the treatment of human disease in the near future. Nanoparticles show potential in effectively removing contaminants and bacteria in water treatment [67] (Table 1). The potential applications of lanthanum calcium manganate nanoparticles (LCMO) include their use in antibacterial drugs and water treatment technologies. These nanoparticles, along with other types such as colossal magneto resistance (CMR) and Eu3+doped lanthanum calcium manganate nanoparticles (LECMO), have the ability to efficiently remove pollutants and microbes from water. The synthesized nanoparticles ranged in size from 50 nm to 200 nm and exhibited a single-phase LCMO or LECMO structure with an orthorhombic crystal structure after annealing the precursor at 1000_C for 2 h in the air. Additionally, the antibacterial activity against *P. aeruginosa*-ATCC 27853 was assessed. The study found that LCMO nanoparticles exhibited superior antibacterial activity compared to LECMO nanoparticles [68]. Cobalt oxide (Co_3_O_4_) nanoparticles have diverse applications in engineering, biomedical, and environmental fields. Co_3_O_4_ nanoparticles and their nanocomposites are utilized to eliminate antibiotic-resistant bacteria in wastewater [69]. Nano-Ag has become increasingly popular as an antibacterial nanomaterial due to its potent and broad antibacterial activity, safety, and ease of production, making it the preferred material for water decontamination [70]. The release of silver ions from nano-silver in water leads to the binding and damage of SH groups in enzyme systems [71]. The antibacterial effect of Nano-silica silver Nano composite (NSAgNC) was investigated for water disinfection against *P. aeruginosa*, demonstrating significant antibacterial activity in a dosing strategy, resulting in the elimination of 99 % of *P. aeruginosa* within 5 h at a concentration of 1.5 mg/mL NSAgNC (5.1 wt% AG). The early binding of NSAgNC to the cell wall damaged cell membrane integrity and led to cytoplasm leakage. The inhibition of respiratory chain dehydrogenase by NSAgNC caused the inactivation of cell metabolism and decreased cell viability [72]. In contrast, iron- and copper-based nanoparticles are anticipated to interact with peroxides in the environment, generating free radicals that are highly toxic to microorganisms. Recently, copper oxide nanoparticles (80–160 nm) were evaluated for their antibacterial activity against *P. aeruginosa*, *Klebsiella pneumonia, Salmonella paratyphoid*, and *Shigella* strains. Additionally, nanoparticles of zinc oxide (ZnO) and magnesium oxide (MgO) are effective in eliminating microorganisms and are currently used as food preservatives [73].

**Table 1:** List of nanoparticles and their minimum bactericidal concentration (MBC) against *P. aeruginosa*.

Name of Nanoparticles	MBC (mg/ml)	References
Ag	2	[74]
Cu	5	[75]
Cu_2_O	2.5	[75]
CuO	5	[75]
ZnO	>5.0	[76]
TiO_2_	8	[76]
La (0.67) Ca (0.33) MnO_3_	5.0–8.0	[68]

### 7.2 Advanced disinfection for waterborne pathogens

Pathogenic microorganisms in water, food, and foam can be effectively eliminated using commonly used disinfectants, including UV and chlorine [77]. Chlorination of drinking water has played a vital role in preventing and controlling waterborne disease epidemics globally [78]. Organic chemicals can undergo various pathways for response, such as electrophilic substitution, addition, and oxidation. The rate constant for chlorination of organic compounds can differ significantly, ranging from less than 0.1 to 10^9^ M^−1^ s ^−1^. On the other hand, chlorination of inorganic chemicals typically happens through an electrophilic attack of HOCl [79]. UV irradiation uses is another disinfection method that utilizes ultraviolet light to damage the DNA of bacteria, viruses, and other pathogens in water, preventing their replication. UV irradiation and chlorination have been used for a long time to disinfect water. However, the use of either UV irradiation or chlorination exclusively may have limitations, such as the potential for live but non-culturable bacteria production and bacterial reactivation [80]. Various methods have been explored to disinfect drinking water and wastewater, including the use of UV and chlorine. In a study, *P. aeruginosa* was selected as the target pathogen to test the feasibility of the UV/chlorine method, and it was found that this method significantly reduced metabolic activity and harmful gene levels in the pathogen [81]. Notably, UV/chlorine therapy was more effective in preventing bacterial dark reactivation than UV or chlorination alone. In addition, advanced UV oxidation processes with hydrogen peroxide and UV photolysis are being investigated to extend their reach to the inactivation of DNA-damaged and antibiotic-resistant bacteria in wastewater treatment [82]. Another study showed that exposure to chlorine led to a decrease in *P. aeruginosa* numbers, but cells acclimated to tap water could no longer be cultured [83]. An ultraviolet (UV)-based advanced oxidation process with hydrogen peroxide and UV radiation was used as a pretreatment method to control biofilms in water. Surviving cells of *P. aeruginosa* were found to develop an adherent biofilm under UV-based Advance oxidation processes (AOP) therapy, while H_2_O_2_/UV could only prevent biofilm formation over long periods in combination with residual H_2_O_2_. The potential benefit of AOP was demonstrated when it resulted in better biofilm management than UV-based AOP therapy maintained with comparable amounts of residual H_2_O_2_ [84] (Table 2). However, the chlorination process was found to promote the natural horizontal transformation of RP4 plasmids, leading to the exchange of ARGs between *P. aeruginosa* and the formation of new ARBs, as well as the transfer of chlorine-damaged opportunistic pathogens from non-ARB to ARB [85]. Additionally, a study on the outcome of *P. aeruginosa* biofilm showed that biofilm extracellular polymeric substances had different effects on biofilm-resistant and detached biofilms on chlorine-based disinfectants, suggesting the importance of understanding the structure and composition of biofilms in developing effective disinfection strategies [86].

**Table 2:** Biofilm formation (%) of *P. aeruginosa* PAO1 after short (<24 h, left) and long (days, right) incubation periods post treatment, using (a) full-UV with 5 mg l-1 H_2_O_2_, and filtered-UV (>295 nm) with (b) 5 mg l-1 H_2_O_2_ and (c) 1 mg l-1 H_2_O_2_. Adapted from Ref. [84].

(a) Full-UV and 5 mg l^−1^ H_2_O_2_	18 h					3 days				
	Control	H_2_O_2_	15s	30s	60s	Control	H_2_O_2_	15s	30s	60s
(1) Control	100-+16					100-+4	78-+6			
(2) H_2_O_2_ 5mg1^−1^										
(3) Full-UV		17-+3	34-+3	27-+2	4-+2			84-+5	82-+3	71-+2
(4) Full- UV + OH (catalase)			40-+8	22-+1	3-+1					
(5) Full- UV + OH + residual H_2_O_2_			3-+1	3-+1	2-+1			6-+10	7-+13	1-+0

(b) Full-UV (>295 nm) and 5 mg l^−1^ H_2_O_2_	22 h					9 days				
	Control	H_2_O_2_	5 min	10 min	15 min	Control	H_2_O_2_	5 min	10 min	15 min
(1) Control	100-+19					100-+7				
(2) H_2_O_2_ 5mg1^−1^		79-+5					100			
(3) Full-UV			71-+5	59-+11	60-+5			100	100	100
(4) Full- UV + OH (catalase)			55-+5	34-+2	18-+2					
(5) Full- UV + OH + residual H_2_O_2_			1-+1	1-+1	0-+0			100	100	2-+2

(c) Full-UV (>295 nm) and 1 mg l^−1^ H_2_O_2_	22 h					9 days				
	Control	H_2_O_2_	5 min	10 min	15 min	Control	H_2_O_2_	5 min	10 min	15 min
(1) Control	100-+19					100-+7				
(2) H_2_O_2_ 5mg1^−1^		87-+4					100			
(3) Full-UV			71-+5	59-+11	60-+5			100	100	100
(4) Full- UV + OH (catalase)			56-+4	39-+6	38-+11					
(5) Full- UV + OH + residual H_2_O_2_			65-+8	39-+6	27-+6			100	100	100

### 7.3 Solar-powered oxidation and ozonation: *P. aeruginosa* and antibiotic resistance genes

Numerous studies have investigated the efficacy of solar-powered Fenton oxidation and ozonation on *P. aeruginosa* and ARGs [87]. Since the 1950s, ozone treatment has been used to treat hospital wastewater, and several studies have demonstrated its effectiveness in eliminating a range of pathogens, including *P. aeruginosa* [88]. Additionally, AOP have been employed to treat water contaminated with diverse pollutants. These techniques are capable of killing microbes due to their potent oxidizing ability and reactivity [68]. The oxidative species, OH, is formed in Fenton reactions by the interaction of Fe^2^ with H_2_O_2_ (Fe_2_ H_2_O_2_/Fe^3^ OH OH). In Fenton reactions powered by sunlight, the Fe^3^ created during the reaction is photo-reduced to regenerate the Fe^2^ (for example, [Fe (H_2_O)]^3^+hv/Fe^2^ H^+^+ OH) [89]. Ozone, as gaseous molecule and allotrope of oxygen, reacts with both inorganic and organic substances, and ozonation is a powerful method for reducing or inactivating microorganisms by generating highly reactive radicals [90]. One mechanism of ozone disinfection involves the disintegration of cell walls, which resulted in the escape of molecules from the cell, and damage to nucleic acids and carbon-nitrogen bonds of proteins [91,92]. The electro-Fenton method was found to be more effective in removing both intracellular and extracellular ARGs of two targets, *tetA* and *ampC*, harbored in *P. aeruginosa*. In the presence of 1.0 mmol/L Fe_2_, after 120 min of electro-Fenton treatment, the removal efficiency was 3.8 log for intracellular tetA, 4.1 log for intracellular ampC, 5.2 log for extracellular tetA, and 4.8 log for extracellular ampC. This method holds promise for removing intracellular and extracellular ARGs from wastewater [93]. Graywater is wastewater generated from bathtubs, showers, sinks, and kitchen sinks, which does not contain sanitary wastewater. In disinfection experiments, adding hydrogen peroxide (150 mg. L1) to pretreated graywater and exposing it to natural sunlight or artificial sunlight from UV lamps resulted in the complete inactivation of *P. aeruginosa* [94].

### 7.4 Advancing photocatalysis and Photodynamic approaches for antibiotic-resistant pathogens

**Fig. 3:**
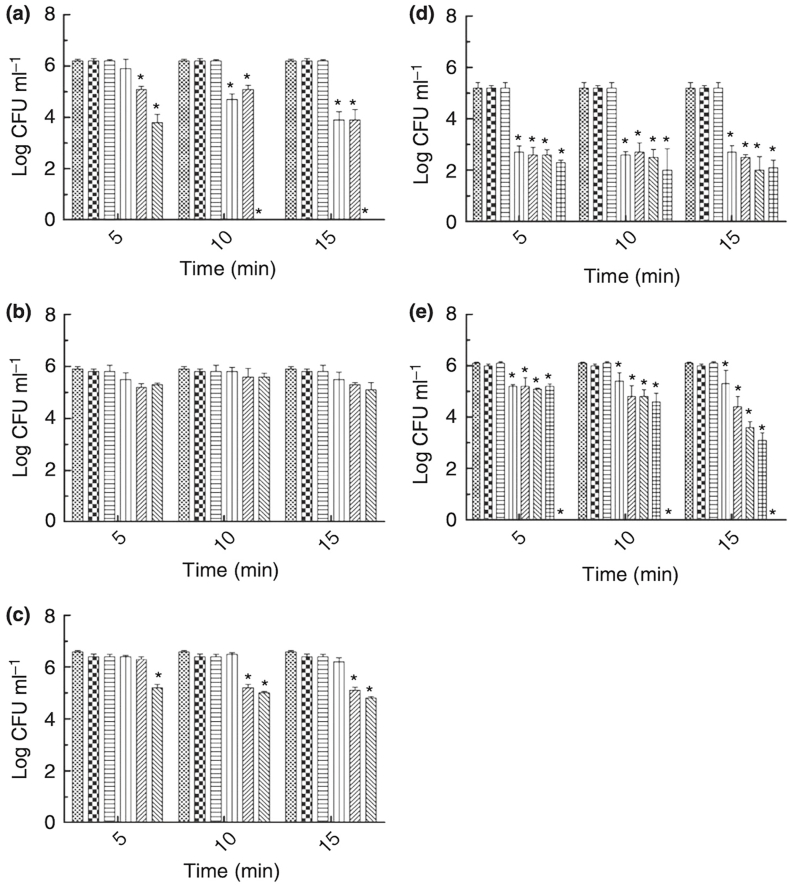
Photodynamic inactivation of bacteria mediated by eosin Y at different concentrations and irradiated by green LED (5, 10 and 15 min). (a) *P. aeruginosa*, (b) *Escherichia coli*, (c) *Salmonella Typhimurium*, (d) *Bacillus cereus* and (e) *Staphylococcus aureus*. Positive control (bacteria and PBS), photosensitizer control (bacteria and eosin Y without light) and light control (bacteria exposed to LED in the absence of photosensitizer). Columns with symbols differ statistically (*P* < 0·05) (a–c:  positive control;  photosensitizer control;  light control;  0·5 μmol l^−1^;  5·0 μmol l^−1^; 10·0 μmol l^−1^. d:  positive control;  photosensitizer control;  light control; 1·0 μmol l^−1^;  2·5 μmol l^−1^;  5·0 μmol l^−1^;  7·5 μmol l^−1^. e:  positive control;  photosensitizer control;  light control;  0·1 μmol l^−1^;  0·25 μmol l^−1^;  0·5 μmol l^−1^;  1·0 μmol l^−1^;  5·0 μmol l^−1^). Adapted from Ref. [49].

Several research papers have been released discussing the photocatalytic inactivation of antibiotic-resistance genes. Additionally, Photodynamic therapy is an emerging approach to combat the pathogenic diseases [49,95] (Fig. 3). The effectiveness of this therapy can be further improved by utilizing chemical penetration that enhance to bacterial membrane permeability. On study used the cationic photosensitizer toluidine blue O (TBO) to inhibit *P. aeruginosa*, and found that the presence of TBO (5 g/mL) significantly reduced bacterial growth [96]. Photocatalysis is a highly advanced method for treating wastewater due to its rapid oxidation, lack of byproducts, and ability to oxidize impurities and hydrolysis byproducts at the parts per billion level [97]. whereas the photocatalytic processes is indirect regarding which reactive oxygen species (O_2_, OH, ^1^ O_2_) are generated (e.g., TiO_2_ + hv + H_2_O --- O_2_, OH, ^1^ O_2_) [98]. Nanotechnology has also yielded promising water treatment such as photocatalysis, nanofiltration, and nanosorbents [99]. Photocatalysis involves a complex five-phase process that includes reactant diffusion, absorption, reaction, desorption and product diffusion [100], and uses light-active nanostructured catalyst media to degrade various water pollutants. In one study, the photocatalytic activity of titanium dioxide (TiO_2_) nanoparticles showed significant antibiofilm activity against *P. aeruginosa* [101,102] and TiO_2_ photocatalysis was found to reduce the two ARGs, *mecA* and *ampC*, in the antibiotic-resistant host bacterium *P. aeruginosa* [103,104].

Treatment with TiO_2_/UVA was found to damage the bacterial membrane and disrupt cell viability and cultivability, as well as suppress the production of virulence factors such as biofilm, protease, and lipase [105]. UVA/TiO_2_ treatment also eliminated *P. aeruginosa's* ability to communicate through “quorum sensing” and mobility with swarm types. Heterogeneous photocatalysis using UVA/TiO_2_ P25 slurry (200 mg L-1 30), UVA/TiO_2_-immobilised and VA/TiO_2_-immobilised/H_2_O_2_ was also effective in inactivating antibiotic-resistant *P. aeruginosa* [106]. The photocatalytic activity of CuO/ZnO inhibited the bacterial growth by 1.8 log CFU/mL upon irradiation with visible light [107]. In another study, Bi_2_WO_6_/BiVO_4_ composites were found to have higher photocatalytic antifouling activity than pure Bi_2_WO_6_ and BiVO_4_ when irradiated with visible light and were able to destroy *P. aeruginosa* after 30 min of [108].

## 8 Conclusion

In summary, *P. aeruginosa* is recognized as harmful species responsible for severe diseases in both humans and animals, exhibiting widespread antibiotic resistance and distribution in nature. Therefore, this review aims to compile information concerning the spread of these bacteria and various strategies for their elimination. The presence of ARGs and ARB has been documented in diverse environments, including sewage treatment plants, wastewater, sewage sludge, municipal solid waste, soils, and lakes, rivers, and livestock operations worldwide. *P. aeruginosa*, naturally found in experimental wastewater, emerges as a promising indicator for wastewater disinfection. However, a research gap persists regarding the efficacy of disinfection methods in real-world drinking water and wastewater treatment plants.

Chemical disinfectants such as chlorine, ozone, and Fenton's reagent demonstrate effectiveness in inactivation *P. aeruginosa* and ARGs but further understanding is needed regarding their ability to remove ARGs from treated water, especially considering the widespread use of chlorination. While low-dose UV radiation shows limited efficacy against conjugative transmission, high-dose UV irradiation in deactivating *P. aeruginosa* and ARGs, albeit with some impact on plasmid-mediated transmissions [109]. TiO2 photocatalytic techniques show promise in activating *P. aeruginosa* and ARGs, although optimization is necessary due to extended treatment durations. Additionally, exploring the role of nanoparticles in wastewater and sewage sludge in influencing ARGs transmission between different genera represents a prospective a venue for research.

In conclusion, this review underscores the widespread presence of ARGs and ARB in various environmental contexts and highlights the potential of *P. aeruginosa* as an innovative indicator for wastewater disinfection. While certain disinfection approaches exhibit promise, further investigation is crucial for a comprehensive understanding of their effectiveness in mitigating ARG spread. Advancements in UV irradiation, photocatalytic methods and nanoparticle research are pivotal in enhancing our capability to combat antibiotic resistance dissemination in aquatic ecosystems.

## Funding

The authors have not received any financial support from funding agencies for this work.

## CRediT authorship contribution statement

**Alariqi Reem:** Writing – review & editing, Writing – original draft, Methodology, Formal analysis, Data curation, Conceptualization. **Siham Almansoob:** Writing – review & editing, Writing – original draft. **Ahmed M. Senan:** Writing – review & editing. **Aditya Kumar Raj:** Writing – review & editing. **Rajesh Shah:** Writing – review & editing. **Mukesh Kumar Shrewastwa:** Writing – review & editing. **Jay Prakash Prasad Kumal:** Writing – review & editing, Writing – original draft, Conceptualization.

## Declaration of competing interest

The authors declare that they have no known competing financial interests or personal relationships that could have appeared to influence the work reported in this paper.
